# Investigation of the Relationship of Some Antihypertensive Drugs with Oxidant/Antioxidant Parameters and DNA Damage on Rat Uterus Tissue 

**Published:** 2011-09-23

**Authors:** Suleyman Salman, Serkan Kumbasar, Nesrin Gursan, Yakup Kumtepe, Bunyamin Borekci, Beyzagul Polat, Hamit Hakan Alp, Mustafa Talip Sener, Halis Suleyman

**Affiliations:** 1 Ministry of Health, Obstetrics and Gynecology Hospital, Igdir, Turkey; 2 Department of Pathology, Ataturk University Medical Faculty, 25240 Erzurum, Turkey; 3 Department of Obstetrics and Gynecology, Ataturk University Medical Faculty, 25240 Erzurum, Turkey; 4 Department of Pharmacology, Ataturk University Medical Faculty, 25240 Erzurum, Turkey; 5 Department of Biochemistry, Ataturk University Medical Faculty, 25240 Erzurum, Turkey; 6 Department of Forensic Medicine, Ataturk University Medical Faculty, 25240 Erzurum, Turkey

**Keywords:** Antihypertensive, Oxidant/Antioxidant Parameters, DNA Damage

## Abstract

**Background:**

In this study, we investigated the effects of treatment with chronic antihypertensive drugs (clonidine, methyldopa, amlodipine, ramipril and rilmenidine) on oxidant-antioxidant parameters and toxic effects on DNA in rat uterus tissue. In addition, uterus tissues were examined histopathologically.

**Materials and Methods:**

A total of 36 albino Wistar rats were divided into the following six groups: 0.075 mg/kg clonidine group; 100 mg/kg methyldopa group; 2 mg/kg amlodipine group; 2.5 mg/kg ramipril group; 0.5 mg/kg rilmenidine group; and the healthy group. Rats underwent chronic drug administration for 30 days and at the end, biochemical and histopathological examinations were performed. All data were subjected to one-way ANOVA test.

**Results:**

We divided these drugs into the following three groups according to their effects on rat uteri: (I) mild negative effects (clonidine), (II) moderate negative effects (rilmenidine, methyldopa) and (III) drugs which had severe negative effects (amlodipine, ramipril).

**Conclusion:**

These data may help with selection of antihypertensive drugs, in order to determine which drugs have the lowest toxicity in pregnant and non-pregnant (pre-pregnancy) women.

## Introduction

The value of anti-hypertensive drugs in treatment of primary and secondary hypertension is well established. Hypertensive disturbances provoke maternal and fetal morbidity and mortality in 10% of pregnancies (1) and is the second highest mortality reason after pregnancy embolus (2). Hypertension is present in the early ages of men and in older women (3). Therefore, various antihypertensive drugs are used for pregnant, hypertensive women. Two or more drug types are generally used in the treatment of hypertension (4, 5).

Toxic effects of drugs may be functional, biochemical, structural and specific. Such effects are evaluated by assessment of oxidant–antioxidant parameters in tissues and severity of DNA damage (6). Oxidants are free oxygen radicals which cause tissue damage (7) by attacking lipids, DNA and proteins, all of which are crucial for living cells (8). Tissue damage initiates with formation of lipid radicals in the cell membrane, continues with conversion to lipid hydroxyperoxides and ends with formation of toxic products such as aldehyde and malondialdehyde (7, 9). There are, however, enzymatic and non-enzymatic defense mechanisms against those toxic products in tissues, called antioxidant systems (9, 10). If defense mechanisms fail to suffice, free radicals induce damage on DNA as well as lipids and proteins, as mentioned above. The product reflecting DNA damage in the blood is 8-hydroxy guanine (8-OH Gua) (11). Studies have proven that 8-OH Gua increases in damaged tissue (6). In a literature review, no results were found on the effects of clonidine, methyldopa, amlodipine, ramipril and rilmenidine on rat uterine tissue. Investigating biochemical effects of antihypertensive drugs on uterine tissue may help in determining the indication and contraindications of these drugs in women.

The aim of our study is to investigate effects of some antihypertensive drugs (clonidine, methyldopa, amlodipine, ramipril and rilmenidine) on oxidant-antioxidant parameters and DNA in rat uterus tissue. We also histopathologically examine the effects of these antihypertensive drugs on uterus tissue.

## Materials and Methods

### Chemicals

Whole biochemical assay compounds were purchased from the following sources: Zdorove Drug, Ukraine (clonidine); Eczacıbaşı Drug, Turkey (methyldopa); Pfizer Drug, Turkey (amlodipine); Aventis Drug, Turkey (ramipril) and Servier Pharmaceuticals, France (rilmenidine).

### Animals

A total of 36 female healthy adult albino Wistar rats that weighed 205-210 g were obtained from the Ataturk University Medicinal and Experimental Application and Research Center. These rats were divided into six treatment groups before the experimental procedures were initiated: 0.075 mg/kg clonidine group, 100 mg/kg methyldopa group, 2 mg/ kg amlodipine group, 2.5 mg/kg ramipril group, 0.5 mg/kg rilmenidine group and healthy group. Animals were housed and fed under standard conditions in a laboratory where the temperature was kept at 22°C. Animal experiments were performed in accordance with National Guidelines for the Use and Care of Laboratory Animals and approved by the local Animal Care Committee of Ataturk University.

### Drug testing

In the experiment, clonidine (0.075 mg/kg), methyldopa (100 mg/kg), amlodipine (2 mg/kg), ramipril (2.5 mg/kg) and rilmenidine (0.5 mg/kg) were administrated to female rats by gastric gavage for 30 days, once a day (12). Distilled water was given to the healthy control group as vehicle, at the same time. After 30 days, all rats were anesthetized by thiopental sodium (25 mg/kg) and blood samples were collected from their hearts. Then, animals were sacrificed under high dose (50 mg/kg) thiopental and their uteri were taken and stored for biochemical analyses in a -80°C deep freezer, and for histopathological analyses in formalin solution.

### Biochemical analyses

#### Malondialdehyde (MDA) analysis

The concentrations of uterus lipid peroxidation were determined by estimating MDA using the thio barbituric acid test (13). Rat uteri were promptly excised and rinsed with cold saline after which they were weighed and homogenized in 10ml of 100 g/L KCl. The homogenate (0.5 ml) was added to a solution that contained 0.2 ml of 80 g/l sodium lauryl sulfate, 1.5 ml of 200 g/l acetic acid, 1.5 ml of 8 g/L 2-thiobarbiturate and 0.3 ml distilled water. The mixture was incubated at 98°C for 1 hour. Upon cooling, 5 ml of n-butanol:pyridine (15:l) was added. The mixture was vortexed for 1 minute and centrifuged for 30 minutes at 4000 rpm. The absorbance of the supernatant was measured at 532 nm. The standard curve was obtained by using 1,1,3,3- tetramethoxypropane.

#### Superoxide dismutase (SOD) analysis

Measurements were performed according to Sun et al. in uterus tissue (14). SOD estimation was based on the generation of superoxide radicals produced by xanthine and xanthine oxidase, which react with nitro blue tetrazolium (NTB) to form purple colored-formazan dye. The sample was centrifuged at 6000 rpm for 10 minutes and then the brilliant supernatant was used as an assay sample. The supernatant was allowed to immediately react with xanthine oxidase. The assay tubes were incubated for one minute and formazan was then measured at 560 nm. In the presence of more enzyme, there will be less O2•¯radical to react with NBT.

#### Glutathione peroxidase (GPx) analysis

GPx activity was determined according to the method of Lawrence and Burk (15). The absorbance at 340 nm was recorded for 5 minutes.

#### Total glutathione (tGSH) analysis

The amount of GSH in the total homogenate was measured according to the method of Sedlak and Lindsay with some modifications (16). The sample was weighed and homogenized in 2 ml of 50mM tris–HCl buffer that contained 20mM EDTA and 0.2mM sucrose at pH 7.5. The homogenate was immediately precipitated with 0.1 ml of 25% trichloroacetic acid and the precipitate was removed after centrifugation at 4200 rpm for 40 minutes at 4°C. The supernatant was used to determine GSH using 5,5-dithiobis (2-nitrobenzoic acid) (DTNB). Absorbance was measured at 412 nm using a spectrophotometer.

#### Isolation and hydrolization of DNA

DNA isolation from blood was performed according to Miller et al. (17) with some modifications. Blood (2 ml) with ethylene diamine tetraacetic acid (EDTA) was mixed with 3 ml of erythrocyte lysis buffer, and incubation for 10 minutes in ice was followed by centrifugation (10 minutes at 1500 x g). The supernatant was decanted and the pellet was resuspended thoroughly in sodium dodecyl sulfate (10%, v ⁄v), proteinase K (20 mg⁄ml) and 1.9 ml leukocyte lysis buffer (4M Nacl and 0.5 M EDTA). The mixture was incubated at 65°C for 1 hour and then mixed with 0.8 ml of 9.5 M ammonium acetate. After centrifugation at 1500 x g for 25 minutes, the clear supernatant (2 ml) was transferred to a new sterile tube and DNA was precipitated by the addition of 4 ml ice-cold absolute ethanol. DNA samples were dissolved in tris EDTA buffer (10 mm, pH=7.4) and then hydrolyzed according to Shigenaga’s method (18).

#### Analysis of 8-OHdG and dG by high-performance liquid chromatography

In the hydrolyzed DNA samples, 8-OHdG and dG levels were measured using high-performance liquid chromatography (HPLC) with electrochemical (HPLC-ECD) and variable wavelength detector (HPLC-UV) systems as described previously (19). Before analysis with HPLC, the hydrolyzed DNA samples were redissolved in the HPLC eluent (final volume 1 ml). Final hydrolysate (20 l) was analyzed by HPLC-ECD (HP, Agilent 1100 modular systems with HP 1049A ECD detector; Agilent, Aldbronn, Germany); and a reverse phase-C18 (RP-C18) analytical column (250 mm × 4.6 mm, 4.01 micron particle size; Phenomenex, Torrance, California, USA). The mobile phase consisted of 0.05 M potassium phosphate buffer (pH=5.5) and acetonitrile (97:3, v/v) with a flow rate of 1 ml/min. The dG concentration was monitored based on absorbance (245 nm) and 8-OHdG based on electrochemical reading (600 mV). Levels of dG and 8-OHdG were quantified using the standards of dG and 8-OHdG from Sigma; 8-OHdG level was expressed as the number of 8-OHdG molecules per 106 dG (20).

#### Histopathological analyses

The specimens were fixed in 10% buffered formalin and routinely processed for paraffin embedding. From each sample, 4 μm thick sections were obtained and stained with hematoxylin eosin to evaluate. The slides were evaluated under light microscopy (Olympus BX51; Olympus Corp., Tokyo, Japan) at 40× magnification. The histopathological evaluation of uterine injury was performed based on parameters that included: degeneration, hemorrhage, edema, vascular proliferation, congestion, neutrophil and eosinophil infiltration. The severity of uterine injury was judged by using a blind semiquantitative scoring system according to previously defined criteria: no injury = 0, mild injury = 1, moderate injury = 2 and severe injury = 3.

### Statistical analyses

All data were subjected to one-way ANOVA using SPSS 13.0 software. Differences between treatment groups and the healthy group were attained using the Scheffe option. Significance was declared at p<0.05 as mean ± SEM.

## Results

### Oxidant and antioxidants results

As in table 1, the following MDA levels were seen: clonidine (0.075 mg/kg), 14.2 ± 0.34; methyldopa (100 mg/kg), 20.8 ± 1.55; amlodipine (2 mg/kg), 25.8 ± 0.94; ramipril (2.5 mg/kg), 41.6 ± 0.98; and rilmenidine (0.5 mg/kg),19.5 ± 0.76 μmol/g. GSH levels were: clonidine (0.075 mg/kg), 9.0 ± 0.4; methyldopa (100 mg/kg), 6.6 ± 0.3; amlodipine (2 mg/kg), 6.1 ± 0.3; ramipril (2.5 mg/kg), 4.2 ± 0.2; and rilmenidine (0.5 mg/kg), 8.1 ± 0.6 U/g. SOD activity was measured as: clonidine (0.075 mg/kg), 6.2 ± 0.35; methyldopa (100 mg/kg), 6.3 ± 0.32; amlodipine (2 mg/kg), 6.8 ± 0.27; ramipril (2.5 mg/kg), 4.5 ± 0.28; and rilmenidine (0.5 mg/ kg), 7.6 ± 0.45 U/g. GPx activity was: clonidine (0.075 mg/kg),11.9 ± 0.73; methyldopa (100 mg/ kg), 7.0 ± 0.47; amlodipine (2 mg/kg), 6.5 ± 0.45; ramipril (2.5 mg/kg), 5.2 ± 0.36; and rilmenidine (05. mg/kg), 8.6 ± 0.86 U/mg. In the healthy rat group, the following values were noted: MDA (11.3 ± 0.26), GSH level (10.6 ± 0.4), SOD (6.4 ± 0.55) and GPx activity (12.5 ± 0.22).

### 8-OH Gua molecules/105 gua molecule analyses

As shown in table 2, the 8-OH Gua/105 Gua levels in the clonidine (0.075 mg/kg), methyldopa (100 mg/kg), amlodipine (2 mg/kg), ramipril (2.5 mg/ kg) and rilmenidine (0.5 mg/kg) groups were: 1.01 ± 0.07, 2.44 ± 0.44, 2.96 ± 0.19, 3.05 ± 0.38 and 2.15 ± 0.10, respectively. This level was 1.21 ± 0.08 in the healthy rat group.

### Histopathological

**Fig 1:**
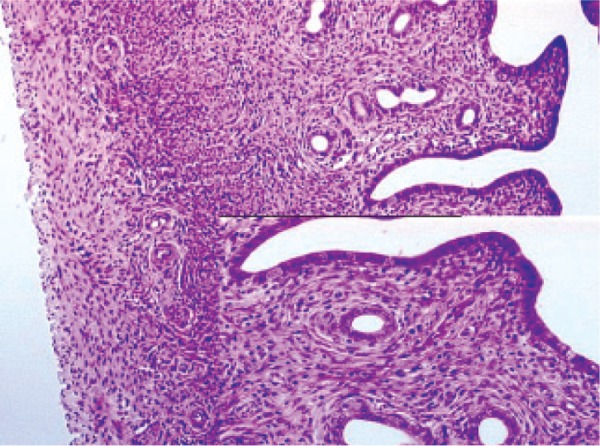
Histopathological examination of clonidine in the rat uterus.

**Fig 2:**
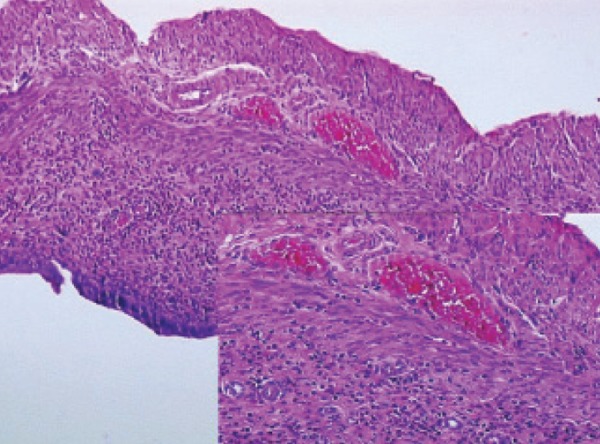
Histopathological examination of amlodipine in the rat uterus.

**Fig 3:**
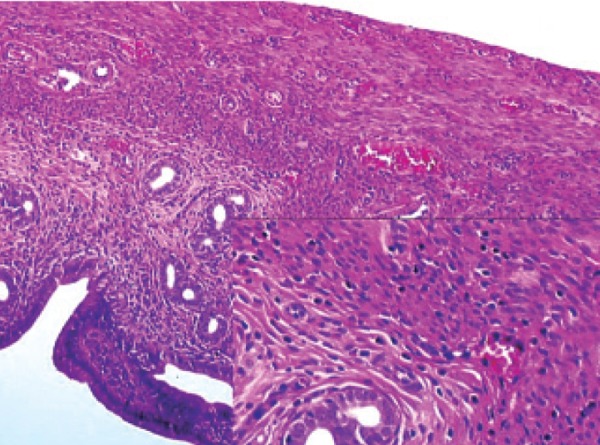
Histopathological examination of rilmenidine in the rat uterus.

**Fig 4:**
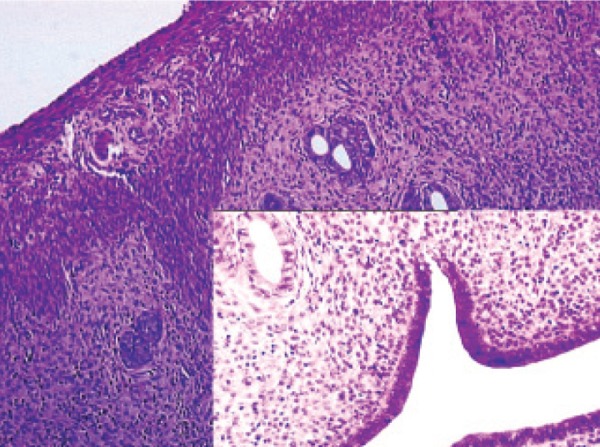
Histopathological examination of methyldopa in the rat uterus.

**Fig 5:**
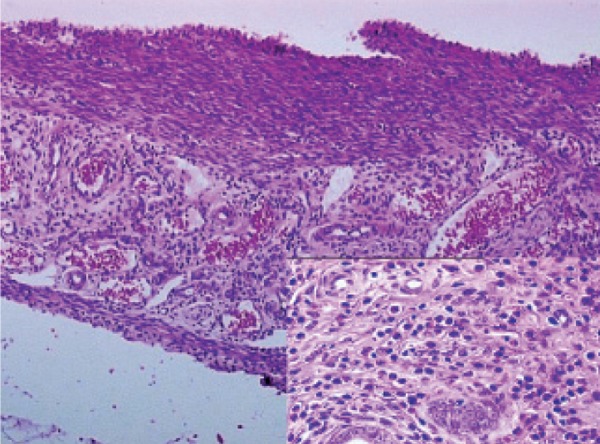
Histopathological examination of ramipril in the rat uterus.

**Fig 6:**
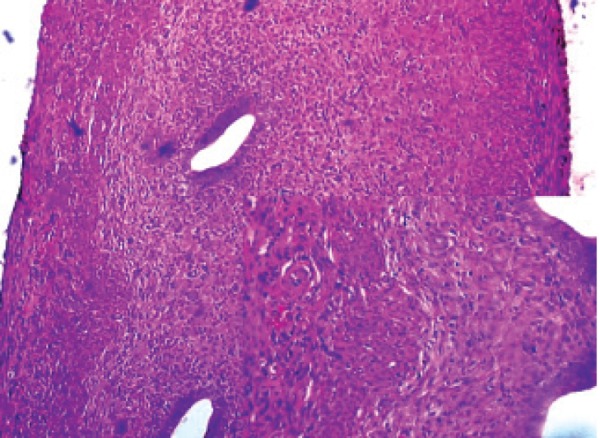
Histopathological examination of healthy rat uterus.

Macroscopically, no alterations occurred in the uteri of the clonidine, methyldopa, amlodipine, ramipril and rilmenidine groups. As seen in table 3 and figures 1-6, microscopic observations of some pathologic parameters such as degeneration, hemorrhage, edema, vascular proliferation, congestion, neutrophil infiltration and eosinophil infiltration showed varying ratios and severities.

**Table 1:** Effect of antihypertensive drugs on oxidant and antioxidant parameters in rat uterine tissue. All the treatment groups were compared with the healthy control group


Drugs	MDA (µmol/g)	p	SOD (U/g)	p	GPx (U/mg)	p	GSH (U/g)	p

**Clonidine (0.075 mg/kg)**	14.2 ± 0.34	>0.05	6.2 ± 0.35	>0.05	11.9 ± 0.73	>0.05	9.0 ± 0.4	>0.05
**Methyldopa (100 mg/kg)**	20.8 ± 1.55	<0.0001	6.3 ± 0.32	>0.05	7.0 ± 0.47	<0.0001	6.6 ± 0.3	<0.0001
**Amlodipine(2 mg/kg)**	25.8 ± 0.94	<0.0001	6.8 ± 0.27	>0.05	6.5 ± 0.45	<0.0001	6.1 ± 0.3	<0.0001
**Ramipril(2.5 mg/kg)**	41.6 ± 0.98	<0.0001	4.5 ± 0.28	<0.05	5.2 ± 0.36	<0.0001	4.2 ± 0.2	<0.0001
**Rilmenidine(0.5 mg/kg)**	19.5 ± 0.76	<0.0001	7.6 ± 0.45	>0.05	8.6 ± 0.86	<0.05	8.1 ± 0.6	<0.05
**Healthy**	11.3 ± 0.26	-	6.4 ± 0.55	-	12.5 ± 0.22	-	10.6 ± 0.4	-


P values represent differences between the treatment groups and healthy control group. Results are expressed as mean±SEM (n=6).

**Table 2:** Effect of antihypertensive drugs on oxidative DNA damage in rat uterine tissue. Treatment groups were compared with healthy control group


Drugs	Dose (mg/kg)	Animals(n)	DNA damage 8-OH Gua/105 Gua pmol/L	P value

**Clonidine**	0.075	6	1.01 ± 0.07	>0.05
**Methyldopa**	100	6	2.44 ± 0.44	<0.05
**Amlodipine**	2	6	2.96 ± 0.19	<0.0001
**Ramipril**	2.5	6	3.05 ± 0.38	<0.0001
**Rilmenidine**	0.5	6	2.15 ± 0.01	<0.05
**Healthy**	-	6	1.21 ± 0.08	-


P values represent the significance between the treatment groups and healthy control group. Results are expressed as mean ± SEM

**Table 3:** Histopathological evaluation of uterine injury based on parameters including degeneration, hemorrhage, edema, vascular proliferation, congestion, neutrophil inﬁltration and eosinophils


	Clonidine	Amlodipine	Rilmenidine	Methyldopa	Ramipril	Healthy

**PNL**	++	++	+++	++	+++	+
**Lymphocyte**	+	++	++	++	+++	0
**Eosinophil**	+	++	+	+	+++	+
**Edema**	+	+++	++	++	+++	0
**Congestion**	+	+++	+	++	+++	0
**Vascular**	+	++	+	+	+++	0
**proliferation**						


The severity of uterus injury was judged by using a blind semiquantitative scoring system according to previously deﬁned criteria: no injury = 0, mild injury = +, moderate injury = ++ and severe injury = +++.

## Discussion

In this study, the effects of antihypertensive drug (clonidine, methyldopa, amlodipine, ramipril and rilmenidine) treatment on oxidant-antioxidant parameters and toxic effects on DNA was investigated in rat uterus tissue. In addition, uterus tissues were examined histopathologically. The reason for choosing these doses is that in a previous study we investigated the effect of these same doses on ovarian tissue. We determined that clonidine (0.075 mg/kg) and rilmenidine (0.5 mg/kg) had no clear negative effect, methyldopa (100 mg/kg) and amlodipine (2 mg/ kg) had negative effects and ramipril (2.5 mg/ kg) had a severe negative effect on ovarian tissue of rats (12). Our study on uterus tissue was in accordance with our previous study on ovarian tissue.

Oxidants are radicals of oxygen origin that induce serious damage in tissues (21, 22). Oxidants easily react with polyunsaturated fatty acids (PFA), amino acids and nucleic acids, causing damage (23). PFA are most affected by oxidants. PFA oxidation is referred to as lipid peroxidation, which results in malondialdehyde (MDA) formation (24). MDA causes transverse binding and polymerization of cell membrane components. This phenomenon disturbs cell structure and function (23). In our study, the MDA level in the clonidine group was found to be almost same as that of the control group. MDA levels in methyldopa, amlodipine and rilmenidine groups were significantly higher than the control group. The highest MDA level was recorded in the group administered ramipril.

Ramipril caused a significantly higher repression of SOD activity than the control group. There was no significant change in SOD activities of clonidine, rilmenidine, methyldopa and amlodipine groups when compared to that of the control. SOD catalyzes conversion of superoxide into peroxide and molecular oxygen (25). Hydrogen peroxide is known to be less harmful than superoxide radicals. SOD is suggested to be a damage-preventing antioxidant enzyme. Studies have shown the ameliorating effects of SOD in burn ulcers and open wounds (26).

No change in GPX activity of uteri of the clonidine rats was recorded. Differences between GPX activities of the clonidine and rilmenidine groups and the control group were found to be statistically insignificant. The most meaningful decrease of GPX activity, when compared to the control group, was observed in the ramipril group. GPX activities of methyldopa and amlopidin groups were significantly repressed when compared to the control group. GPX activity was found to be lower in damaged tissue than undamaged tissue, and shown to be proportional to the severity of damage (6).

There was no change in GSH levels in the uteri of the clonidine group. Differences in GSH activity between clonidine and rilmenidine, and control groups were statistically insignificant. The lowest GSH level was observed in the ramipril group. Previous studies have demonstrated that GSH levels decrease in damaged tissue (27-29). GSH possesses a protective effect via converting harmful hydrogen peroxide to harmless water (30). GSH is known to protect protein tiol groups, which are important for protection and longevity of cell integrity, against oxidation (31). In a previous work, we have shown that estrogen and luteinizing hormone (LH) prevent gastric damage by stimulating α_2_-adrenergic receptors (32). In addition, estrogen and LH have been shown to repress oxidant parameters and stimulate antioxidant parameters in stomach tissue; it is understood that estrogen and LH produce this antioxidant effect via α_2_-adrenergic receptors (33). The antioxidant effect of estrogen and LH in uterus tissue has been reported to vanish upon treatment with the α_2_-adrenergic receptor blocker yohimbine (34). These data suggest that α_2_-adrenergic receptors are responsible for cytoprotection, not only in the stomach but also in uterine tissue.

Clonidine is a selective agonist of α_2_-adrenergic receptors, while rilmenidine is rather a selective agonist of imidazolin l1 receptors. Lack of any negative effects on uterine tissue by clonidine may be attributed to stimulation of α_2_-adrenergic receptors by clonidine. Amongst other drugs in this study (methyldopa, amlodipine, ramipril), the least toxic on uterine tissue was rilmenidine. This phenomenon may also be due to an agonist effect of rilmenidine on α_2_-adrenergic receptors. However, the agonist of α_2_-adrenergic receptors, methyldopa, was found to repress MDA less than clonidine and rilmenidine, but repressed GSH more than clonidine and rilmendin. These negative effects of methyldopa may be attributed to inhibition of dopadecarboxylase enzyme, which converts methyldopa to alpha-methyl-noradrenalin (active metabolite) in noradrenergic neuron terminals. Inhibition of this enzyme hinders formation of the active metabolite (alpha-methylnoradrenalin) of methyldopa.

Oxygen radicals may generate toxic effects on DNA. Hydroxyl radical causes DNA damage by removing hydrogen from nucleic acids and reacting with double bonds (35). 8-hydroxyguanin (8-OH GUA) is a product of DNA damage and accepted as an indicator of DNA damage in blood (11, 36).

Under normal circumstances, there is a balance of oxidative damage and repair in DNA. This shows that, although very little, there is DNA damage in healthy individuals, too (37). In our study, 8-OH GUA levels in the uteri of the clonidine group were lower than that of healthy controls, although the difference was statistically insignificant. The 8-OH GUA amount in the rilmenidine group was higher than that of the control group, but the difference was insignificant. These two drugs (clonidine and rilmenidine) were found to have the lowest negative effects on uterine tissue and blood DNA, when compared to other drugs (methyldopa, amlodipine and ramipril) used in study.

The biochemical toxic effects of these drugs on uterus tissue were supported via histopathologic examination which determined the structural toxic effects of these drugs. Microscopic examinations showed that histopathologic parameters such as degeneration, hemorrhage, edema, vascular proliferation, congestion, neutrophil infiltration and eosinophil infiltration were seen the least in the clonidine group and most in the ramipril group. The degree of these histopathological data correlated with biochemical toxic effects. PNL and lymphocyte infiltration show the severity of drug infiltration (38).

## Conclusion

We divided these drugs into three groups according to their effects on the uterus: (I) mild negative effects (clonidine); (II) moderate negative effects (rilmenidine, methyldopa); and (III) severe negative effects (amlodipine, ramipril). These data may help in the selection of antihypertensive drugs that have the lowest toxicity in pregnant and non-pregnant (pre-pregnancy) women.

## References

[1] Sibai BM (1996). Treatment of hypertension in pregnant women. N Engl J Med.

[2] North RA, Taylor RS, Schellenberg JC (1999). Evaluation of a definition of pre-eclampsia. Br J Obstet Gynaecol.

[3] Crawford MH, DiMarco JP, Paulus WJ (2010). Crawford Cardiology.

[4] Black HR, Elliott WJ, Neaton JD, Grandits G, Grambsch P, Grimm RH Jr (2001). Baseline Characteristics and Early Blood Pressure Control in the CONVINCE Trial. Hypertension.

[5] Cushman WC, Ford CE, Cutler JA, Margolis KL, Davis BR, Grimm RH (2002). Success and predictors of blood pressure control in diverse North American settings: the antihypertensive and lipid-lowering treatment to prevent heart attack trial (ALLHAT). J Clin Hypertens (Greenwich).

[6] Polat B, Suleyman H, Alp HH (2010). Adaptation of rat gastric tissue against indomethacin toxicity. Chem Biol Interact.

[7] Naito Y, Yoshikawa T, Yoshida N, Kondo M (1998). Role of oxygen radical and lipid peroxidation in indomethacininduced gastric mucosal injury. Dig Dis Sci.

[8] Halliwell B, Aruoma OI (1991). DNA damage by oxygenderived species.Its mechanism and measurement in mammalian systems. FEBS Lett.

[9] Anderson D (1996). Antioxidant defences against reactive oxygen species causing genetic and other damage. Mutat Res.

[10] Bast A, Haenen GR, Doelman CJ (1991). Oxidants and antioxidants: state of the art. Am J Med.

[11] Grollman AP, Moriya M (1993). Mutagenesis by 8-oxoguanine: an enemy within. Trends Genet.

[12] Salman S, Kumbasar S, Yilmaz M, Kumtepe Y, Borekci B, Bakan E (2011). Investigation of the effects of the
chronic administration of some antihypertensive drugs
on enzymatic and non-enzymatic oxidant/antioxidant
parameters in rat ovarian tissue. Gynecol Endocrinol.

[13] Ohkawa H, Ohishi N, Yagi K (1979). Assay for lipid peroxides in animal tissues by thiobarbituric acid reaction. Anal Biochem.

[14] Sun Y, Oberley LW, Li Y (1988). A simple method for clinical assay of superoxide dismutase. Clin Chem.

[15] Lawrence RA, Burk RF (1976). Glutathione peroxidase activity in selenium-deficient rat liver. Biochem Biophys Res Commun.

[16] Sedlak J, Lindsay RH (1968). Estimation of total, proteinbound, and nonprotein sulfhydryl groups in tissue with Ellman's reagent. Anal Biochem.

[17] Miller SA, Dykes DD, Polesky HF (1988). A simple salting out procedure for extracting DNA from human nucleated cells. Nucleic Acids Res.

[18] Shigenaga MK, Aboujaoude EN, Chen Q, Ames BN (1994). Assays of oxidative DNA damage biomarkers 8-oxo- 2'-deoxyguanosine and 8-oxoguanine in nuclear DNA and biological fluids by high-performance liquid chromatography with electrochemical detection. Methods Enzymol.

[19] Floyd RA, Waston JJ, Wong PK, Altmiller DH, Rickard RC (1986). Hydroxy free radical adduct of deoxyguanosine: Sensitive detection and mechanisms of formation. Free Radic Res Commun.

[20] Kasai H (1997). Analysis of a form of oxidative DNA damage, 8-hydroxy-2'-deoxyguanosine, as a marker of cellular oxidative stress during carcinogenesis. Mutat Res.

[21] Halliwell B (1994). Free radicals, antioxidants, and human disease: curiosity, cause, or consequence?. Lancet.

[22] Halliwell B, Gutteridge JM (1984). Oxygen toxicity, oxygen radicals, transition metals and disease. Biochem J.

[23] Freeman BA, Crapo JD (1982). Biology of disease: free radicals and tissue injury. Lab Invest.

[24] Comporti M (1985). Lipid peroxidation and cellular damage in toxic liver injury. Lab Invest.

[25] Fridovich I (1995). Superoxide radical and superoxide dismutases. Annu Rev Biochem.

[26] Zamora Rodriguez ZB, Gonzalez Alvarez R, Guanche D, Merino N, Hernandez Rosales F, Menendez Cepero S (2007). Antioxidant mechanism is involved in the gastroprotective effects of ozonized sunflower oil in ethanol-induced ulcers in rats.Mediators Inflamm. Mediators Inflamm.

[27] Cadirci E, Suleyman H, Aksoy H, Halici Z, Ozgen U, Koc A (2007). Effects of Onosma armeniacum root extract on ethanol-induced oxidative stress in stomach tissue of rats. Chem Biol Interact.

[28] Dengiz GO, Odabasoglu F, Halici Z, Suleyman H, Cadirci E, Bayir Y (2007). Gastroprotective and antioxidant effects of amiodarone on indomethacin-induced gastric ulcers in rats. Arch Pharm Res.

[29] Suleyman H, Cadirci E, Albayrak A, Polat B, Halici Z, Koc F (2009). Comparative study on the gastroprotective potential of some antidepressants in indomethacin- induced ulcer in rats. Chem Biol Interact.

[30] Martensson J, Meister A (1991). Glutathione deficiency decreases tissue ascorbate levels in newborn rats: ascorbate spares glutathione and protects. Proc Natl Acad Sci USA.

[31] Stein HJ, Hinder RA, Oosthuizen MM (1990). Gastric mucosal injury caused by hemorrhagic shock and reperfusion: protective role of the antioxidant glutathione. Surgery.

[32] Borekci B, Kumtepe Y, Karaca M, Halici Z, Cadirci E, Albayrak F (2009). Role of alpha-2 adrenergic receptors in anti-ulcer effect mechanism of estrogen and luteinising hormone on rats. Gynecol Endocrinol.

[33] Kumtepe Y, Borekci B, Karaca M, Salman S, Alp HH, Suleyman H (2009). Effect of acute and chronic administration of progesterone, estrogen, FSH and LH on oxidant and antioxidant parameters in rat gastric tissue. Chem Biol Interact.

[34] Borekci B, Ingec M, Kumtepe Y, Karaca M, Koc F, Salman S (2009). Effect of estrogen, progesterone, LH and FSH on oxidant and antioxidant parameters in rat uterine tissue. International Journal of Fertility and Sterility.

[35] Milligan JR, Ward JF (1994). Yield of single-strand breaks due to attack on DNA by scavenger-derived radicals. Radiat Res.

[36] Cadet J, Douki T, Gasparutto D, Ravanat JL (2003). Oxidative damage to DNA: formation, measurement and biochemical features. Mutat Res.

[37] Collins AR, Dusinska M, Gedik CM, Stetina R (1996). Oxidative damage to DNA: do we have a reliable biomarker?. Environ Health Perspect.

[38] Hinkle AJ, Weinlander CM (1989). The effects of 10% methohexital on the rectal mucosa in mice. Anesthesiology.

